# The association of internet use and cognition among older adults: mediating roles of social networks and depressive symptoms

**DOI:** 10.3389/fpsyt.2025.1569022

**Published:** 2025-04-24

**Authors:** Xiuyuan Zhu, Zihan Ni, Xiaoying Zhu, Yi Yang, Shiyu Xie, Xinguo Wang, Xiaoguang Yang

**Affiliations:** ^1^ School of Elderly Care Services and Management, Nanjing University of Chinese Medicine, Nanjing, China; ^2^ Nossal Institute for Global Health, School of Population and Global Health, The University of Melbourne, Melbourne, VIC, Australia; ^3^ School of Public Health, Fudan University, Shanghai, China; ^4^ College of Public Health, Shanghai University of Medicine & Health Sciences, Shanghai, China; ^5^ Chinese Hospital Development Institute, Shanghai Jiaotong University School of Medicine, Shanghai, China

**Keywords:** older adults, internet use, social networks, depressive symptoms, cognition

## Abstract

**Background:**

To explore the chain-mediated roles of social networks and depressive symptoms in the relationship between internet use and cognition among older adults.

**Methods:**

Data were sourced from the 2018 China Longitudinal Aging Social Survey (CLASS). Structural equation modeling (SEM) was employed to analyze the potential mediating roles of social networks and depressive symptoms. Internet use, Lubben Social Network Scale (LSNS), the Center for Epidemiological Studies Depression Scale (CES-D) and Cognitive Scale were selected as indicators. Additionally, propensity score matching (PSM) was applied to further validate the robustness of the results by reducing potential selection bias and ensuring a more comparable distribution of observed covariates between internet users and non-users.

**Results:**

Our analysis found that the use of the internet by older adults was positively correlated with their social networks (*β*=0.090, *p*<0.01). Both internet use and social networks exhibited negative associations with depressive symptoms in older adults respectively (*β*=-0.244, *p*<0.01; *β*=-0.136, *p*<0.01). Furthermore, both internet use (*β*=0.177, *p*<0.01) and social networks (*β*=0.032, *p*<0.01) positively predicted cognition in older adults, while depressive symptoms (*β*=-0.090, *p*<0.01) negatively impacted cognition. Moreover, our study showed that internet use not only directly influenced cognition of older adults but also indirectly impacted it through social networks and depressive symptoms, yielding a total mediated effect value of 0.026. Social networks and depressive symptoms accounted for 1.48% and 10.84% of the total effect respectively. Social networks and depressive symptoms served as chain mediators between internet use and cognition, constituting 0.49% of the total effect.

**Conclusion:**

This chain-mediated model provided a clear depiction of how social networks and depressive symptoms mediate the impact of internet use on the cognition of older adults. Enhancements in internet literacy and optimization of age-appropriate product designs are recommended to improve cognitive functions in older populations.

## 1 Introduction

With the intensification of global aging, the decline in cognitive abilities among older adults has emerged as an urgent public health issue (1). Cognitive impairment in older adults can lead to significant declines in physical and mental health and daily functioning, manifesting as decreased activities of daily living (2), increased frailty (3), emotional disorders (4) and a reduced quality of life (5). Studies indicate that approximately 15% of Chinese aged 60 and above suffer from mild cognitive impairment (6), with this percentage increasing to 30% among individuals aged 80 and older (7). The severest form of cognitive impairment, such as dementia, leads to significant disability among older adults, with more than 24 million affected worldwide—about a quarter of whom are in China, where approximately 300,000 new cases occur each year (8). Given the critical role of cognitive health in the overall well-being of older adults, exploring effective intervention measures holds significant practical importance.

In recent years, the advent and prevalence of the internet provided the older adults with new avenues for cognitive stimulation and social interaction (9). Recent data indicate a significant uptick in older adults engaging with the internet via smartphones, computers, tablets, and other devices. According to the 50th Statistical Report on Internet Development in China, in 2022, the country’s internet users topped 1.051 billion, with those aged 60 and above comprising 11.3%, a figure that continues to grow rapidly (10). The role of internet use in maintaining and improving cognitive abilities in older adults has been confirmed (11). As internet-based information technologies like smartphones and wearables become more integrated into daily cognitive activities, older individuals may develop enhanced online cognitive capabilities, potentially surpassing those of younger generations (12). Furthermore, the clinical implications of using video games for cognitive training in older adults are profound, offering potential interventions for cognitive decline (13). Although existing studies suggest that internet use has a potential positive impact on the cognitive functions of the elderly, the specific mechanisms are not yet fully understood.

The internet serves not only as a platform for information acquisition and entertainment (13, 14) but also as a pathway for older adults to engage in social activities. Existing research suggested that broader social networks (8) and reduced depressive symptoms (15) are strongly linked to cognitive improvements in the older people. However, aging is often accompanied by social isolation (16) and depression (17), influenced by diminished financial resources (18) and lack of social support (19) may mitigate these issues by enriching social interactions and enhancing psychological well-being, indirectly benefiting cognitive health (20). Nevertheless, the adverse effects of depression include impacts on brain vasculature, neuroendocrine metabolism, and the immune system (21, 22). Therefore, when assessing the impact of social networks on the cognitive abilities of older adults, it is essential to account for the potential influence of depressive emotions. Current research lacked a detailed explanation of the complex interactions among these variables, warranting further investigation. This study aims to uncover the mechanisms by which internet use, social networks, and depressive symptoms influence cognition in older adults. We hypothesize that internet use positively affects cognition by enhancing social networks and alleviating depressive symptoms. The outcomes of this research will not only enrich theoretical frameworks regarding older adults’ internet usage and cognitive health but also inform the design of effective internet-based interventions to promote cognitive well-being and overall quality of life among the older adults.

### 1.1 The mediating role of social networks

The advent of the internet has increased the avenues through which older adults can build their social networks (23). As a dynamic web, the internet provides older individuals with efficient communication channels, enhancing their ability to stay connected with family and friends, thereby stabilizing their social networks (9). Through the internet, older individuals can transcend the limitations of time and space, fostering connections with new individuals, engaging in meaningful online interactions, and participating in offline activities. This expansion and enrichment of social networks potentially increase their social capital (8). Social capital is defined as the resources obtainable through social connections, including personal or community relationships (e.g., social networks, reciprocal norms, etc.) (24). It is broadly categorized into two dimensions: psychological/cognitive and network/structural (25). On one hand, psychological capital includes elements such as trust, reciprocity, adherence to group norms, and perceptions of the social environment. On the other hand, network social capital pertains to resources gained through social networks (25), typically measured by social participation and the dissemination of information (26).

The expansion of social networks has demonstrable effects on the cognitive abilities of older adults. Epidemiological research has highlighted that being integrated into social networks benefits the health and longevity of adults (27, 28). Older adults, who face heightened health and survival challenges, increasingly rely on these networks to meet their physical, social, and emotional needs (29). There are individual differences in the social networks possessed by older adults, with larger and more diverse networks associated with slower cognitive decline (30, 31). Given the links between internet use, social networks, and cognition, the following hypothesis 1 is proposed:

Hypothesis 1: Social networks would mediate the relationship between internet use and cognition in older adults.

### 1.2 The mediating role of depressive symptoms

Depressive symptoms are significant concern among older adults in China, with nearly 40% reporting symptoms of depression (32). The internet can ameliorate these symptoms in various ways, contributing positively to the emotional well-being of this demographic. For instance, the internet offers diverse leisure activities such as online games, video streaming, and online shopping (33), which can enhance the mood and overall happiness of older adults. Additionally, the internet serves as a resource for health-related information (34), allowing older individuals to manage their physical and mental health proactively. As individuals age, self-stereotypes among older adults tend to solidify, leading to negative expectations for personal development and a consequent decline in mental health (35). However, acquiring information technology skills and utilizing the internet proficiently can reintegrate active older adults into the labor market (36), reaffirming their societal value and mitigating the adverse effects of social isolation and diminished self-esteem.

The internet, as an effective medium for alleviating depressive symptoms among older adults, can enhance cognition by improving their psychological well-being (37, 38). Given the established link between depressive symptoms and cognitive decline—where depressive symptoms often precede and exacerbate cognitive deterioration, particularly affecting memory recall (39), there is a critical need to establish intervention mechanisms for depression among the older adults. The internet presents a viable medium for such interventions (40), although research in this area remains sparse and warrants further exploration. Based on the interconnections between internet use, depressive symptoms, and cognition in older adults, the following hypothesis 2 is proposed:

Hypothesis 2: Depressive symptoms would mediate the relationship between internet use and cognition in older adults.

### 1.3 Multiple mediating effects of social networks and depressive symptoms

The use of the internet has significant potential in expanding social networks, which in turn can alleviate depressive symptoms among older adults. Depressive symptoms in older adults are often linked to inadequate family support, limited social contact, poor physical health, and social isolation (41). As physical conditions gradually deteriorate, older adults may experience deepening depression due to the diminishing size of their social networks. The widespread availability of the internet provides a means for older adults to communicate and interact with others without physical barriers, thus helping to maintain and even expand their existing social networks. Due to special historical, political, and economical factors, China has a significant number of “empty-nest” older adults, a term referring to older adults who live separately from their children, either alone or with a spouse (42). These individuals lack sufficient family and social support, making them particularly susceptible to depression. The use of digital technologies such as the internet can help these individuals enhance their social interactions, accumulate more social capital, and improve their mental health.

The broaden-and-build theory of positive emotions suggests that expanding social networks through the internet not only contributes to the psychological health of older adults but also significantly enhances their cognitive abilities. Positive emotions can broaden individuals’ thought processes and cognitive abilities, enhance cognitive flexibility, widen the scope of attention, and foster cognitive innovation, encouraging higher levels of creativity and problem-solving capabilities in cognitive tasks (43). Therefore, the internet’s role in sustaining and broadening social networks among older adults not only enhances their mental health but also bolsters their cognitive functions. Considering the interplay among internet use, social networks, depressive symptoms and cognition in older adults, the following hypothesis 3 is articulated:

Hypothesis 3: Social networks and depressive symptoms could sequentially mediate the relationship between internet use and cognition in older adults.

## 2 Methods

### 2.1 Data sources

The study used data from the 2018 China Longitudinal Aging Social Survey (CLASS), conducted by Renmin University of China (RUC). CLASS aims to systematically collect comprehensive data on personal demographics, family composition, socioeconomic background, and health status of older adults in China. The survey’s objective is to understand the complexities and challenges faced by older individuals during their aging process, thereby providing essential insights for addressing age-related issues in China. The survey is administered across 28 provinces, autonomous regions, and municipalities. By the completion of the follow-up in 2018, the sample included 11,419 respondents from 476 village committees or neighborhood committees in 30 villages across China.

**Figure 1:**
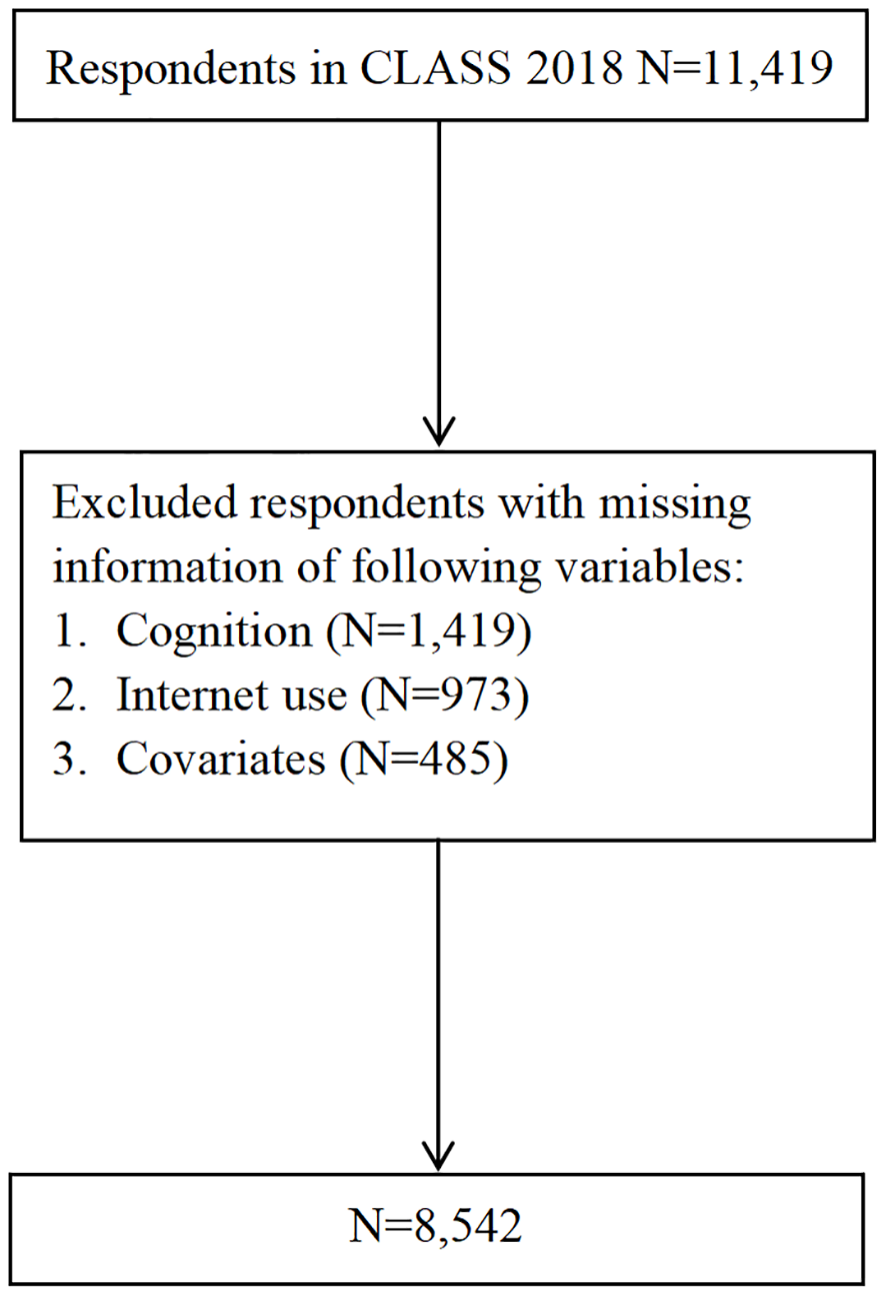
Respondents’ flow in the study.

Given that the CLASS 2018 dataset included numerous variables relevant to this study, such as internet use, social networks, depressive symptoms, and cognition, it was selected as a foundational tool for this research. The study focused on participants aged 60 years and older. After excluding records with incomplete information or missing values for the key variables, the final sample size comprised 8,542 individuals. Among excluded respondents, 1,419 were missing information about cognition and 1,458 were missing for depressive symptoms. The sample selection process was shown in **Figure 1**.

### 2.2 Measurement

#### 2.2.1 Cognition

The dependent variable in this study was the cognition of older people. The CLASS 2018 survey utilized a modified version of the Telephone Interview for Cognitive Status (TICS-m) combined with the Mini-Mental State Examination (MMSE) (44) to evaluate cognitive performance. This scale has since been extensively adopted for screening cognitive impairment among older adults in China (45, 46), covering a variety of cognitive domains including orientation, memory, attention, numeracy, and recall. The instrument comprised 16 questions, awarding one point for each correct answer and zero for incorrect responses, allowing for a total score range from 0 to 16. A higher score on this scale indicated better cognitive ability. The Cronbach’s α coefficient for the scale was 0.72, demonstrating a satisfactory level of internal consistency. This suggested that the individual items within the scale collectively measure a cohesive cognitive construct, thereby strengthening the reliability of the results.

#### 2.2.2 Internet use

In this study, the independent variable was the comprehensive assessment of internet use among older adults, covering several key aspects: frequency of use, type of access devices, proficiency in using these devices, and the purposes of internet use, corresponding to A1 - A4 respectively. The frequency of internet use was assessed on a scale from 0 to 4, where “0” indicated “never using the internet” and “4” represented “using the internet every day.” This categorization helped in understanding the regularity of internet interaction among older adults. CLASS 2018 encompassed three types of internet access devices: smartphones, computers, and tablet PCs (Pad/Ipad). The study calculated scores based on the number of devices used by older individuals, ranging from 0 to 3. For proficiency of device use, the study evaluated on a scale from 0 (Not proficient at all) to 4 (Very proficient), with the total possible score extending up to 12 points for proficiency across all devices. Higher scores indicated a greater mastery over device usage. The study further assessed the purposes of internet use among older people. The study identified 12 different purposes for which the internet was used, including voice or video chats, text messaging, online shopping, consuming news, reading articles beyond news, engaging with music/radio/videos, gaming, managing transportation, health management, financial investments, educational activities, and other uses. Each purpose was given a score of 1 if used, allowing a range from 0 to 12 based on the diversity of use. The Cronbach’s α coefficient for these four dimensions was 0.89, exceeding the threshold of 0.7, indicating good reliability of these items. The discriminant validity criterion required factor loadings above 0.5, significant p-values, composite reliability (CR) above 0.6, and average variance extracted (AVE) above 0.5. The factor loadings for the mentioned dimensions ranged from 0.882 to 0.945, all of which were significant at the level of *p*<0.01. CR was 0.96, and AVE was 0.86, indicating good convergent validity of the latent variables.

#### 2.2.3 Social networks

The CLASS 2018 survey employed the Lubben Social Network Scale (LSNS) (47) to assess the social networks of older adults, focusing on their connections with both family and friends. This scale contained 6 items, measuring the extent to which older adults have relatives and friends they can meet or contact, confide in about personal matters, and depend on for assistance. The responses were categorized into six levels: “none”, “1”, “2”, “3-4”, “5-8”, and “9 and above”, which were assigned scores from 0 to 5 respectively. Consequently, the total possible score on this scale ranged from 0 to 30, where higher scores represented larger social network sizes for the older adults. The Cronbach’s α coefficient for this scale was 0.87, indicating excellent internal consistency. This suggested that the scale reliably captures the social network characteristics of older adults, enhancing the validity of the measurement. The high reliability also reinforced the robustness of the study’s findings, ensuring that variations in social network scores accurately reflect individual differences rather than measurement inconsistencies.

#### 2.2.4 Depressive symptoms

In the study, depressive symptoms among older adults were assessed using the Center for Epidemiological Studies Depression Scale (CES-D) (44) provided in the CLASS 2018 survey. This scale included nine items that capture a range of emotional states and physiological symptoms associated with depression. Specifically, the scale measured six negative emotions and physiological manifestations: loneliness, sadness, loss of appetite, sleep disturbance, feelings of worthlessness, and boredom. It also evaluated three positive emotions: cheerfulness, satisfaction with life, and joyfulness. Each item on the CES-D was scored using a 3-point scale: “never” scoring 0, “sometimes” scoring 1, and “often” scoring 2. The items representing positive emotions were reverse-scored to align with the scale’s focus on depressive symptoms. The total score was calculated by summing the scores of all nine items, resulting in a composite index ranging from 0 to 18. A higher total score indicated a greater level of depressive symptoms among the surveyed older adults. The Cronbach’s α coefficient for the scale was 0.73, indicating a good level of internal consistency among the items. This implied that the scale effectively captures depressive symptoms in older adults, enhancing the credibility of the measurement. The sample items of the above scale can be found in the **Supplementary Materials**: Scale Information.

#### 2.2.5 Control variables

The control variables selected for this study included gender (1=male, 0=female), age (years), education level (0=illiterate, 1=private school/literacy class, 2=primary school, 3=junior high school, 4=high school and above), work (0=unemployed, 1=employed), hukou (household registration status, which distinguishes between rural and urban residency) (0=agricultural, 1=non-agricultural).

### 2.3 Data analysis

The study conducted a comprehensive analysis using SPSS 26.0, encompassing the descriptive analysis, common method bias test, reliability assessment, and correlation analysis among variables. Then the study utilized AMOS 26.0 to examine the mediating mechanism of social networks and depressive symptoms. Structural equation modeling (SEM) assumed causal relationships among a set of latent variables, each represented by linear combinations of observable variables. It not only elucidated the influence of independent variables on dependent variables but also unraveled path dependency and structural relationships among the variables.

However, while SEM and chain mediation analysis provide sophisticated tools for examining the relationships between variables, they inherently rely on the directional hypotheses established at the outset. This approach, combined with the use of cross-sectional data, prevents the assessment of causal directions over time. For instance, although our study suggests that internet use might enhance cognition through expanding social networks, it is equally plausible that individuals with higher cognitive abilities are more likely to engage in digital technology use. Similarly, while depressive symptoms may mediate the relationship between social networks and cognitive function, they could also result from cognitive decline. To mitigate these concerns, this study further conducted robustness tests. Our study applied propensity score matching (PSM) to balance observed covariates between internet users and non-users, thereby enhancing the comparability of groups and validating the robustness of the results. By employing PSM, we aimed to reduce selection bias and provide more reliable estimates of the relationship between internet use and cognitive function in older adults.

This study used several fit indices to assess the goodness of fit of the model, including comparative fit index (CFI; good fit>0.90), goodness of fit index (GFI; good fit>0.90), normed fit index (NFI; good fit>0.90), Tucker-Lewis index (TLI; good fit>0.90), root mean square error of approximation (RMSEA; acceptable fit<0.08), and standardized root mean square residual (SRMR; acceptable fit<0.08).

## 3 Results

### 3.1 Common method bias

The study employed Harman’s single-factor analysis method to analyze the items incorporated in the CLASS 2018. The result identified five common factors with eigenvalues exceeding 1. The first factor accounted for 30.23% of the variance, which was below the threshold of 40%, indicating negligible concerns regarding common method biases.

### 3.2 Descriptive statistics and the partial correlation analysis

The sample comprised 8,542 participants, balanced between genders, with males representing 49.2% and females 50.8%. The average age was 71.1 years. Education levels were generally low; 64.7% had not progressed beyond junior high school, 24.4% completed junior high school, and 10.9% had higher education. A majority, 88.1%, were not living alone, and 76.8% were not currently employed. The distribution between agricultural and non-agricultural households was even, with 50.8% and 29.2% respectively. Regarding lifestyle, 29.4% were smokers and 70.6% non-smokers. Internet use was limited, with 80.5% never using the internet, 16.2% using one device, and 3.3% using multiple devices. Only 7.9% used the internet for more than three purposes.

**Table 1** presented means, standard deviations, and correlation coefficients. Older adults showed limited proficiency in using internet devices, with a mean value of 0.96. The average social network size was 13.67, with a standard deviation of 5.40, which indicated moderate social networks with significant individual variations. The average depression score was 6.64, suggesting low overall depressive symptoms. The mean cognition score was 13.44, reflecting generally good cognitive function among participants.

**Table 1:** Descriptive statistics of variables and partial correlation analysis(N=8542).

Variable	M (SD)	1	2	3	4	5	6	7
1 Frequency of Internet use	0.70 (1.47)	1						
2 Internet access devices	0.24 (0.53)	0.859***	1					
3 Proficiency of device use	0.96 (2.25)	0.830***	0.873***	1				
4 Purposes of Internet use	0.65 (1.53)	0.832***	0.772***	0.758***	1			
5 Social networks	13.67 (5.40)	0.077***	0.037***	0.056***	0.037***	1		
6 Depressive symptoms	6.64 (3.10)	-0.174***	-0.151***	-0.171***	-0.188***	-0.137***	1	
7 Cognition	13.44 (3.01)	0.056***	0.051***	0.039***	0.046***	0.025**	-0.071***	1

**, and *** indicate significant at the 10%, 5%, and 1% levels, respectively.

Correlation analysis indicated positive associations between cognition and internet usage, including frequency of internet use (r=0.056, *p*<0.01), internet access devices (r=0.051, *p*<0.01), proficiency of device use (r=0.039, *p*<0.01), and purposes of internet use (r=0.046, *p*<0.01), as well as social networks (r=0.025, *p*<0.05). Conversely, there was a negative correlation between depressive symptoms and cognition (r=-0.071, *p*<0.01), suggesting that higher depressive symptoms are associated with lower cognitive function.

### 3.3 Examination of the multiple mediation model

**Figure 2:**
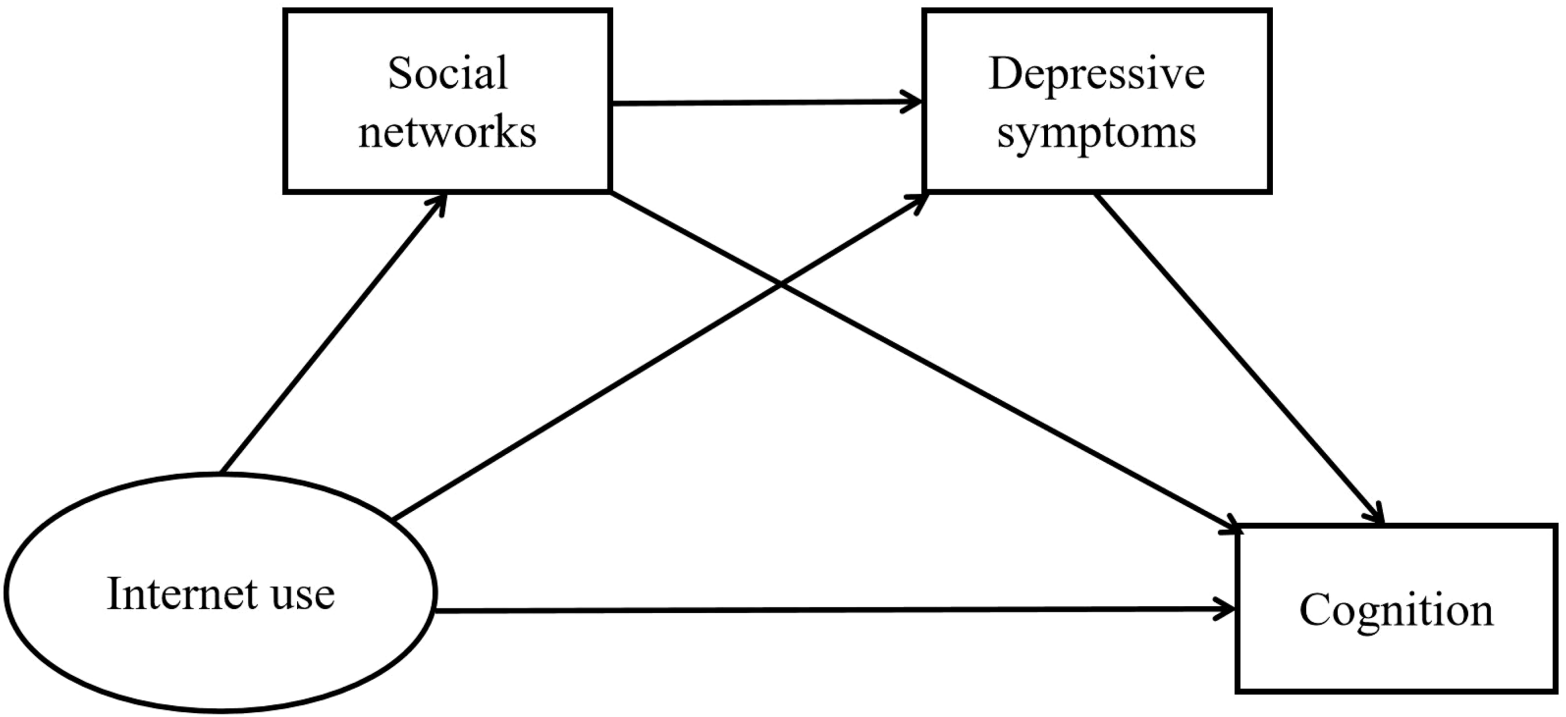
The mechanism of Internet use on cognition among older adults.

The study developed a structural equation model to explore the mediating roles of social networks and depressive symptoms in the relationship between internet use and cognition, as depicted in **Figure 2**. Model fit was assessed using AMOS 26 and multiple fit indices were reported. To ensure the robustness of the proposed chain mediation model, it was compared against five competing models: two simple mediation models, a serial mediation model, a parallel mediation model, and a mixed mediation model. Model comparisons were conducted using multiple fit indices, and the results consistently indicated that the chain mediation model exhibited the best fit. Detailed model comparison information can be found in the **Supplementary Materials**: Model comparison information. This finding suggested that the sequential influence of social networks and depressive symptoms plays a crucial role in explaining the relationship between internet use and cognition, providing a more comprehensive and nuanced understanding than alternative mediation structures.

The chi-square to degrees of freedom ratio (CMIN/DF) for the adjusted model was 21.01, which exceeded the conventional fit threshold of 3. However, this elevated ratio primarily reflected the sensitivity of CMIN/DF to large sample size, which increased the likelihood of rejecting the null hypothesis and resulted in a higher value. Consequently, supplementary fit indices were considered. To enhance the robustness of the evaluation, this study also incorporated information criteria such as the Akaike Information Criterion (AIC) and the Bayesian Information Criterion (BIC). The default model recorded an AIC of 208.615 and a BIC of 349.670. In comparison, the saturated model showed an AIC of 56.000 and a BIC of 253.477, while the independence model exhibited significantly higher values. These results demonstrated that the current model not only significantly outperformed the independence model but also maintained a favorable balance between model complexity and data fit. The relatively low AIC and BIC values further supported the model’s superiority, suggesting that it is an optimal choice among alternative models. Furthermore, the model demonstrated excellent fit, with a GFI of 0.994, CFI of 0.996, NFI of 0.996, IFI of 0.996, TLI of 0.990, RMSEA of 0.048, and SRMR of 0.010, all of which satisfied the standard criteria. These findings further validated the stability and appropriateness of the proposed multiple mediation model.

The structural equation model was estimated using the maximum likelihood method. The mediation effects within the model were tested through the bias-corrected percentile bootstrap method with a 95% confidence interval, using a random sample size of 5,000. A mediation effect is considered significant if the confidence interval does not include zero (48). As detailed in **Table 2**, the use of the internet by older adults was positively correlated with their social networks (*β*=0.090, *p*<0.01). Both internet use and social networks exhibited negative associations with depressive symptoms in older adults respectively (*β*=-0.244, *p*<0.01; *β*=-0.136, *p*<0.01). Furthermore, both internet use (*β*=0.177, *p*<0.01) and social networks (*β*=0.032, *p*<0.01) positively predicted cognition in older adults, while depressive symptoms (*β*=-0.090, *p*<0.01) negatively impacted cognition.

**Table 2:** Analysis of direct effects for SEM.

Outcome variable	Predictive variable	R^2^	β	SE	t	LLCI	ULCI
Equation 1							
Social networks	Internet use	0.008	0.090^***^	0.044	7.765	0.068	0.113
Equation 2							
Depressive symptoms	Internet use	0.084	-0.244^***^	0.025	-21.110	-0.264	-0.224
	Social networks		-0.136^***^	0.006	-13.095	-0.157	-0.116
Equation 3							
Cognition	Internet use	0.051	0.177^***^	0.026	14.519	0.160	0.194
	Social networks		0.032^***^	0.006	3.018	0.009	0.053
	Depressive symptoms		-0.090^***^	0.011	-8.198	-0.114	-0.067

*** indicate significant at the 10%, 5%, and 1% levels, respectively.

As shown in **Table 3**, Internet use not only directly influenced older adults' cognition but also indirectly affected it through social networks and depressive symptoms, yielding a total mediating effect value of 0.026. When examined separately, social networks and depressive symptoms accounted for indirect effects of 0.003 and 0.022 respectively, representing 1.48% and 10.84% of the total effect. This confirmed the support for both Hypothesis 1 and Hypothesis 2. Moreover, social networks and depressive symptoms served as chain mediators between internet use and cognition, with an indirect effect value of 0.001, constituting 0.49% of the total effect, thus affirming Hypothesis 3. The 95% confidence intervals for these indirect effects did not include zero, suggesting that all three indirect effects are statistically significant.

**Table 3:** Analysis of intermediary effects for SEM.

Effect types	Effect	Boot SE	Boot LLCI	Boot ULCI	Ratio of indirect to total effect	Ratio of indirect to direct effect
Total effect	0.203	0.008	0.188	0.219		
Direct effect	0.177	0.008	0.160	0.194		
Total indirect effect	0.026	0.003	0.020	0.033	12.81%	14.69%
Internet use → Social networks → Cognition	0.003	0.001	0.001	0.005	1.48%	1.69%
Internet use → Depressive symptoms → Cognition	0.022	0.003	0.016	0.028	10.84%	12.43%
Internet use → Social networks → Depressive symptoms → Cognition	0.001	0.000	0.001	0.002	0.49%	0.56%

### 3.4 Robustness test

To enhance the robustness of the research conclusions, this study implemented the Propensity Score Matching (PSM) method. By matching the experimental group with a control group sharing similar background characteristics based on propensity scores, we effectively mimicked random assignment of interventions. This approach allowed for better matching of the experimental and control group samples. Subsequently, we used the PROCESS macro in SPSS to conduct a chain mediation analysis.

In our study, propensity scores were calculated using a logit model with five selected covariates. The nearest neighbor matching method with a caliper was applied to match samples from the experimental and control groups. After matching, a total of 5,802 samples were obtained, including 1,616 in the experimental group and 4,186 in the control group. The quality of the matching results was then evaluated using a balance hypothesis test. Following propensity score matching, the standard deviation of covariates between the treatment and control groups was considered optimal if it remained within 10%; values outside this range indicated suboptimal matching quality. As shown in **Table 4**, the standard deviations of most matched variables were significantly reduced, and the absolute bias after matching remained within the 10% threshold, indicating a good fit. Moreover, in the balance test, the *p*-values after matching failed to reject the null hypothesis at the 1% significance level, suggesting no significant differences between the groups after matching.

**Table 4:** Balance diagnose.

Variable	Unmatched	Mean			%reduct	t-test	
	Matched	Treated	Control	%bias	|bias|	t	p>|t|
Gender	U	0.521	0.505	3.3		1.22	0.221
	M	0.522	0.518	0.8	74.8	0.24	0.808
Age	U	66.825	72.102	-83.9		-28.09	0.000
	M	66.837	66.904	-1.1	98.7	-0.38	0.707
Education level	U	2.863	1.723	100.4		34.44	0.000
	M	2.861	2.873	-1.1	98.9	-0.37	0.713
Work	U	0.193	0.241	-11.7		-4.20	0.000
	M	0.191	0.187	1.0	91.3	0.31	0.757
Hukou	U	0.784	0.421	79.9		27.80	0.000
	M	0.784	0.786	-0.4	99.5	-0.13	0.899

Next, we used the PROCESS macro in SPSS to examine the mediating effects among internet use, social networks, depressive symptoms, and cognition. The results were presented in **Additional File**: **Supplementary Table S1**, **S2**. As shown in **Supplementary Table S1**, the three models indicated that after controlling for five covariates, internet use is positively associated with social networks. Additionally, both internet use and social networks negatively predicted depressive symptoms, while positively influencing cognitive function. Conversely, depressive symptoms negatively affected cognition in older adults. In **Supplementary Table S2**, the 95% confidence intervals for the three indirect effects did not include zero, confirming the presence of significant mediation effects. These findings further reinforced the robustness of the SEM results in this study.

## 4 Discussion

The study constructed a multiple mediation model to elucidate the association between internet use and cognition, focusing on the mediating roles of social networks and depressive symptoms among older adults. The results underscored three indirect pathways linking internet use to cognition in older individuals, which indicated that internet usage helped older adults maintain and expand their social networks, which in turn reduces depressive symptoms and boosts cognition.

The finding of the research illustrated the significant role of social networks as a mediator in the relationship between internet use and cognition. Specifically, internet use among older adults facilitates the maintenance and expansion of their social networks, consequently contributing to cognitive improvement. Research has shown that the brain exhibits plasticity in response to environmental stimuli, leading to structural changes in neural circuits (49–51). The internet, as a complex network system, provides opportunities to access various information resources and communication platforms, enabling users to connect with others and establish social networks (41). These networks enhance opportunities for social interaction, allowing older adults to engage with individuals and various groups so as to access various new information and perspectives, as well as to engage in continuous thinking expression and communication. This cognitive stimulation encourages older adults to engage in activities such as logical reasoning, problem-solving, and decision-making (8). This change may significantly enhance the strength of signals in brain regions responsible for decision-making and complex reasoning, potentially positively affecting the responsiveness of neural circuits in older adults’ brains and thereby enhancing their cognitive abilities (52).

The study also unveiled that internet use impacts the cognitive abilities of older adults through the mediating role of depressive symptoms. Depression is a common mental disorders in older adults and is a risk factor for cognitive decline (53).Severe depressive symptoms are associated with poorer cognitive performance (54, 55), potentially due to increased cortisol levels and resulting dysregulation of the hypothalamic-pituitary-adrenal axis and hippocampal atrophy (56). However, the internet provides diverse activities that can mitigate depressive symptoms and enhance cognitive performance, such as watching videos, online shopping, and gaming. These activities can enrich the daily lives of older adults, bringing joy and fulfillment (57, 58), contributing to psychological satisfaction, mental pleasure, and helping them to alleviate depressive symptoms (33). Beyond mood regulation, internet use may influence cognition through neurobiological pathways related to neuroplasticity and cognitive reserve. Engaging in cognitively stimulating activities online, such as reading news, participating in discussions, and solving puzzles can promote synaptic plasticity and enhance cognitive reserve, thereby delaying cognitive decline (59, 60). Specifically, frequent engagement in problem-solving activities online has been linked to increased activation in the prefrontal cortex, a region critical for executive function and working memory (61, 62). Age-related cognitive decline is associated with prefrontal cortex atrophy (63); however, enhancing mood through positive engagement triggers notable activation in this region and boosts dopamine secretion, a neurotransmitter known for its role in neuroplasticity (15). Moreover, internet use may facilitate functional connectivity within key neural networks, such as the default network (DN) and frontoparietal network, which are essential for cognitive processing and memory retention (64, 65). Additionally, mastering internet skills has become increasingly essential for employment in the digital age. Older adults who acquire digital competencies may improve their chances of remaining in or re-entering the job market (36), which can enhance their sense of self-worth and social identity, thereby reducing anxiety and depressive symptoms associated with aging.

Moreover, the outcome of the study illustrated that social networks and depressive symptoms play a chain-mediating role between internet usage and the cognitive ability of older adults. Based on the social exclusion theory, older adults face multidimensional social exclusion due to the transformations in social roles and the compression of social network, including emotional relationship exclusion and public life exclusion (66, 67). This exclusion not only accelerates the loss of social capital (68) but also triggers depressive symptoms and cognitive decline, further driving its malignant chain-like progression. Internet use can increase the online social participation through social network sites and chat groups, effective in building social connections and capital (69, 70), thereby breaking this progression. On one hand, by utilizing social platforms and instant messaging tools to overcome physical space limitations, older individuals can enhance the frequency and quality of communication with family members in different locations (71). This digital intergenerational interaction helps compensate for emotional exclusion in reality and strengthens strong family ties. On the other hand, through interest-based communities, online volunteer activities, and so on, the internet enables older individuals to shift from passive social withdrawal to active social reconstruction (72), establishing weak connections with heterogeneous individuals outside their close circles (73). This form of online social capital accumulation not only compensates for the contraction of offline social networks but also helps them re-integrate into the social structure through information sharing and identity reshaping. The resulting composite social capital has a significant protective effect on psychological health (74), alleviating the loneliness caused by family alienation through strong ties and enhancing a sense of social belonging through weak ties to reduce the risk of depression. According to the broaden-building theory of positive emotion (43), this improvement in psychological well-being can enhance cognitive flexibility and neural plasticity, ultimately breaking the lock-in effect of social exclusion on cognitive function.

To sum up, this research revealed that internet usage has a positive effect on expanding social networks, alleviating depression and improving cognitive abilities of older adults. Based on this, two key recommendations are proposed. Firstly, while expanding internet access, it is crucial to complement it with systematic digital literacy training. Governments and community organizations should implement free or subsidized programs that cover essential digital skills, online safety, and the use of social and health-related applications. Public libraries, senior activity centers, and universities can serve as learning hubs, fostering peer support where experienced older users assist newcomers in adapting to the digital world. Additionally, partnerships with telecommunications providers can offer affordable internet plans tailored for seniors, reducing financial barriers and promoting digital inclusion, ultimately enabling older adults to fully integrate into the digital society. Secondly, the establishment of age-friendly digital interface standards should be promoted to improve the usability of internet products. The government and industry should work together to create age-appropriate design guidelines, ensuring digital products meet the needs of older users. This includes simplifying navigation processes, offering clear interface guidance, and optimizing font sizes and high-contrast color schemes. Additionally, personalized settings should be provided, enabling users to adjust screen brightness, text size, and voice assistance features according to their preferences. The widespread use of accessible technologies, such as voice control, will also help older individuals with mobility limitations use internet products more easily. To further enhance digital inclusivity for older adults, the government can encourage businesses to develop age-friendly products through financial incentives or age-friendly certification mechanisms. Finally, the deep integration of digital technology with the elderly care system should be promoted, making the internet an essential tool for health management and social support. The government should vigorously develop smart care technologies and encourage communities and care institutions to promote digital services such as telemedicine, online health monitoring, and AI assistants. Additionally, communities can establish online volunteer platforms to encourage digital volunteers to help older adults with internet usage issues, enhancing their digital inclusion. By embedding internet technologies into home care, institutional care, and community health service systems, we can improve the accessibility and efficiency of elderly care services significantly. Through the implementation of these measures, older adults will be able to more actively integrate into the digital society, enjoy the benefits of the internet, and improve their mental health and cognitive abilities.

Nonetheless, this study has limitations primarily due to its methodological and data constraints, which limit the ability to explore potential reverse causality. Given these constraints, our study can identify correlations but cannot definitively determine causal pathways or rule out the potential for reverse causality. To address these gaps, future research should not only adopt a longitudinal design but also consider alternative methodologies such as experimental designs or time-series analysis, which can provide more definitive evidence of causation by manipulating variables over controlled periods. These approaches will allow for the observation of how relationships between variables develop over time and can more accurately establish the sequence of events. Longitudinal studies, combined with these alternative methodologies, provide a more robust framework for identifying causal relationships and exploring the dynamic interactions among internet use, social networks, depressive symptoms, and cognitive function. Such studies will not only help validate and expand upon the findings of this study but also enable a clearer understanding of the directionality of the relationships observed.

## 5 Conclusion

With the promotion of healthy aging, there is a growing global focus on positively addressing the challenges of an aging population. China, facing significant demographic shifts due to aging, is increasingly utilizing internet technology to tackle age-related issues. This study summarized previous researches and empirically examined the impacts of internet use on the cognition of older adults using micro data from CLASS 2018. The findings indicated that internet use can enhance cognitive ability by maintaining and broadening older adults’ social networks as well as mitigating depressive symptoms. The results enrich existing literature by providing a new perspective on the relationship between internet use and cognition among older adults, offering significant practical implications for enhancing their well-being.

## Data Availability

Data are available from the China Longitudinal Aging Social Survey (CLASS) (http://class.ruc.edu.cn) for researchers who meet the criteria for access to CLASS data.
