# Supplementary material for: The association of internet use and cognition among older adults: mediating roles of social networks and depressive symptoms
Source: Front Psychiatry. 2025 Apr 24;16:1569022. doi: 10.3389/fpsyt.2025.1569022 (PMC12058668; doi:10.3389/fpsyt.2025.1569022)
Supplement: Supplementary file 1 [file SupplementaryFile1.docx]

Figure S1-S5 Five competing models used for comparison

1.Simple mediation model 1


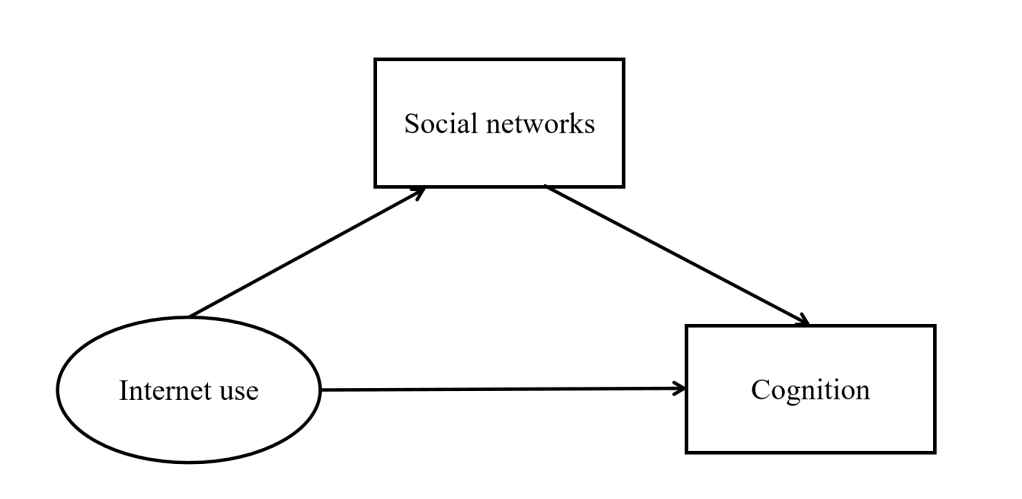


Figure S1 Simple mediation model 1

Partial model fit indices are as follows:

CMIN/DF = 109.040

BIC = 890.020

AIC = 791.282

RMSEA = 0.112

SRMR = 0.046

GFI = 0.972

CFI = 0.982

NFI = 0.981

IFI = 0.982

TLI = 0.960

2.Simple mediation model 2


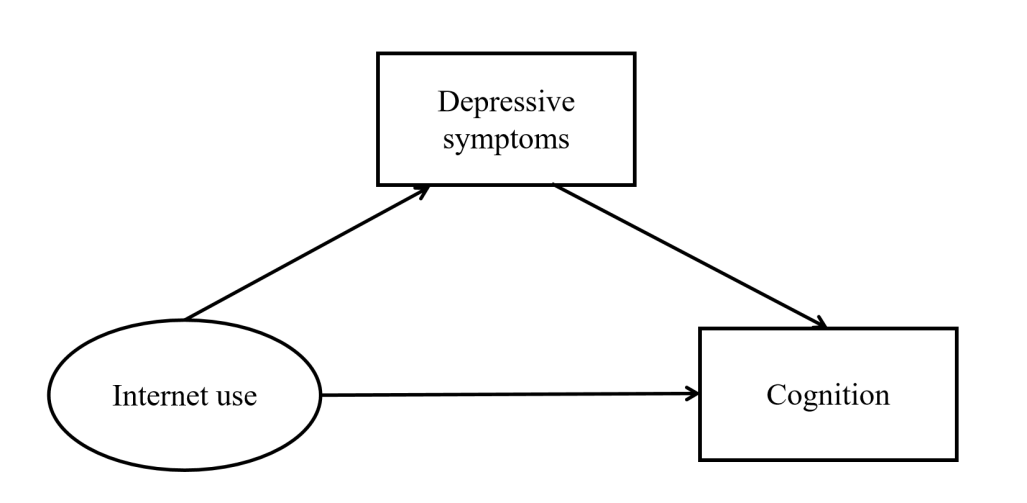


Figure S2 Simple mediation model 2

Partial model fit indices are as follows:

CMIN/DF = 111.683

BIC = 908.523

AIC = 809.784

RMSEA = 0.114

SRMR = 0.047

GFI = 0.971

CFI = 0.981

NFI = 0.981

IFI = 0.981

TLI = 0.960

3.Series mediation model


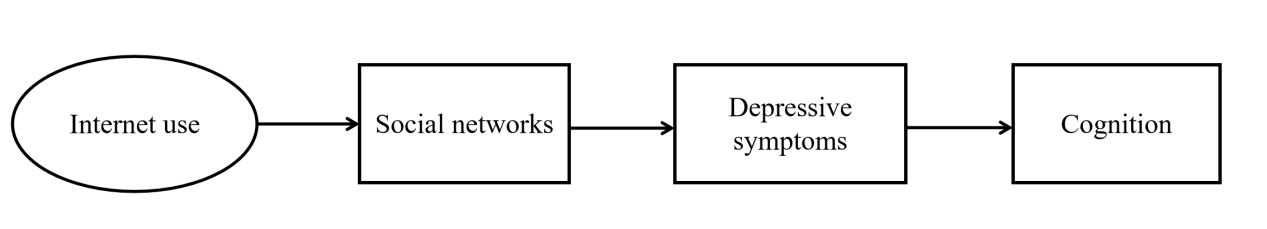


Figure S3 Series mediation model

Partial model fit indices are as follows:

CMIN/DF = 78.535

BIC = 1087.264

AIC = 974.42

RMSEA = 0.095

SRMR = 0.561

GFI = 0.971

CFI = 0.978

NFI = 0.977

IFI = 0.978

TLI = 0.961

4.Parallel mediation model


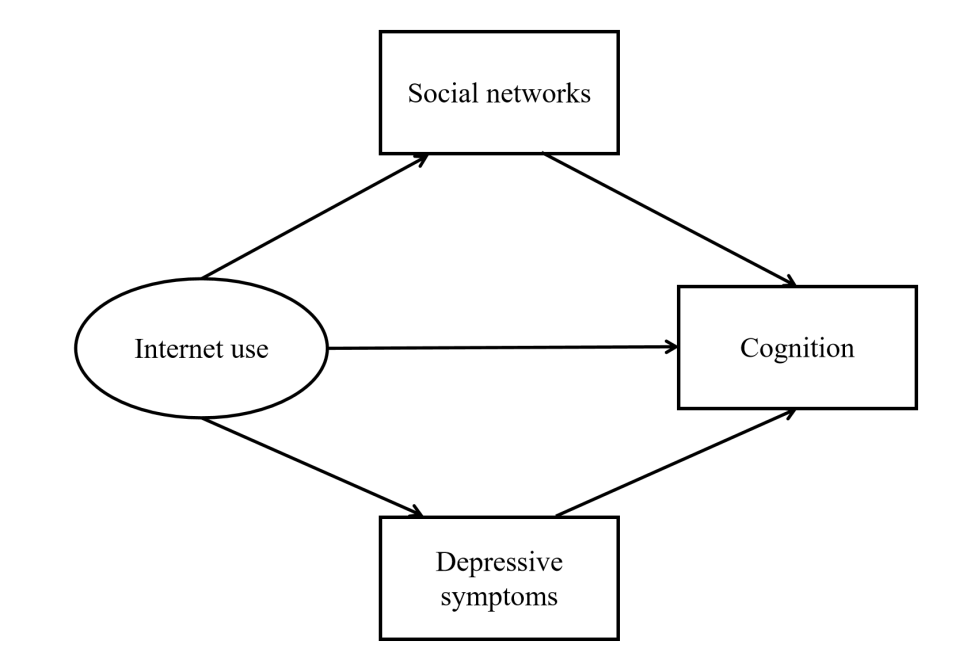


Figure S4 Parallel mediation model

Partial model fit indices are as follows:

CMIN/DF = 33.895

BIC = 427.375

AIC = 279.267

RMSEA = 0.062

SRMR = 0.403

GFI = 0.992

CFI = 0.994

NFI = 0.994

IFI = 0.995

TLI = 0.983

5.Mixed mediation model


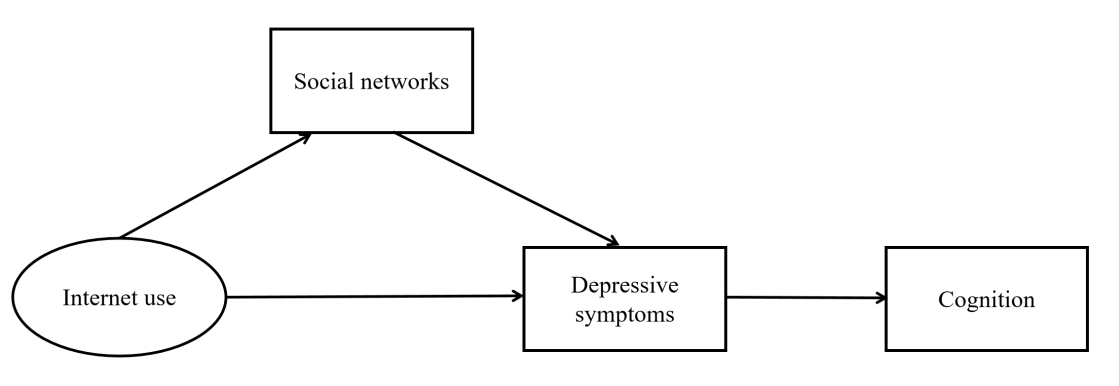


Figure S5 Mixed mediation model

Partial model fit indices are as follows:

CMIN/DF = 45.140

BIC = 542.176

AIC = 401.121

RMSEA = 0.072

SRMR = 0.312

GFI = 0.988

CFI = 0.992

NFI = 0.991

IFI = 0.992

TLI = 0.978
