# Supplementary material for: The association of internet use and cognition among older adults: mediating roles of social networks and depressive symptoms
Source: Front Psychiatry. 2025 Apr 24;16:1569022. doi: 10.3389/fpsyt.2025.1569022 (PMC12058668; doi:10.3389/fpsyt.2025.1569022)
Supplement: Supplementary file 2 [file Table1.docx]

Table S1 Analysis of direct effects

| Outcome variable | Predictive variable | R^2^ | β | SE | t | LLCI | ULCI |
| --- | --- | --- | --- | --- | --- | --- | --- |
| Equation 1 |  |  |  |  |  |  |  |
| Social networks | Internet use | 0.017 | 1.108^***^ | 0.945 | 6.565 | 0.778 | 1.440 |
| Equation 2 |  |  |  |  |  |  |  |
| Depressive symptoms | Internet use | 0.097 | -1.283^***^ | 0.095 | -13.535 | -1.469 | -1.097 |
|  | Social networks |  | -0.083^***^ | 0.007 | -11.232 | -0.097 | -0.068 |
| Equation 3 |  |  |  |  |  |  |  |
| Cognition | Internet use | 0.085 | 0.427^***^ | 0.081 | 5.251 | 0.268 | 0.586 |
|  | Social networks |  | 0.013^**^ | 0.006 | 2.143 | 0.001 | 0.026 |
|  | Depressive symptoms |  | -0.046^***^ | 0.011 | -4.135 | -0.068 | -0.024 |

Table S2 Analysis of intermediary effects

| Effect types | Effect | Boot SE | Boot LLCI | Boot ULCI |
| --- | --- | --- | --- | --- |
| Total effect | 0.078 | 0.017 | 0.045 | 0.113 |
| Internet use → Social networks → Cognition | 0.015 | 0.007 | 0.001 | 0.030 |
| Internet use → Depressive symptoms → Cognition | 0.059 | 0.016 | 0.029 | 0.091 |
| Internet use → Social networks → Depressive symptoms → Cognition | 0.004 | 0.001 | 0.002 | 0.007 |
