# Supplementary material for: The association of internet use and cognition among older adults: mediating roles of social networks and depressive symptoms
Source: Front Psychiatry. 2025 Apr 24;16:1569022. doi: 10.3389/fpsyt.2025.1569022 (PMC12058668; doi:10.3389/fpsyt.2025.1569022)
Supplement: Supplementary file 3 [file Table2.docx]

**Cognition:**

**1. What is today's date? (Both lunar and Gregorian calendars are acceptable)**
Correct.......................................................................... 1

False.............................................................................. 2

Cannot answer.............................................................. 9

**2. What is the name of this neighborhood/village? (Community committee or neighborhood/village name)**
Correct.......................................................................... 1

Fault.............................................................................. 2

Cannot answer.............................................................. 9

**3. When is National Day? (October 1st)**
Correct.......................................................................... 1

Fault.............................................................................. 2

Cannot answer.............................................................. 9

**4. Who is the current President of China? (Xi Jinping)**
Correct.......................................................................... 1

Fault.............................................................................. 2

Cannot answer.............................................................. 9

**5. What is the Chinese zodiac year for this year? (Year of the Dog) [Or: What is this year's zodiac sign?]**
Correct.......................................................................... 1

Fault.............................................................................. 2

Cannot answer.............................................................. 9

**6. I will now say three words, please listen carefully: Apple / Table / Coin. Please repeat them back.**
(Order does not matter; record the number of correct words repeated.)
Correct words: [____]

**7. If you have 100 yuan and spend 7 yuan, how much do you have left? (93 yuan)**
[ | ] yuan

**7.1 If you spend another 7 yuan, how much remains? (86 yuan)**
(If the previous answer was incorrect, do not count this as correct even if the response is now accurate.)
[ | ] yuan

**7.2 If you spend another 7 yuan, how much remains? (79 yuan)**
(If the participant forgets the previous answer, remind them; however, this response will not be counted as correct. Future correct answers based on the previous incorrect one will be counted.)
[ | ] yuan

**7.3 If you spend another 7 yuan, how much remains? (72 yuan)**
(If this calculation is correct, count it as correct.)
[ | ] yuan

**7.4 If you spend another 7 yuan, how much remains? (65 yuan)**
(If this calculation is correct, count it as correct.)
[ | ] yuan

**8. Please repeat the three words I told you earlier.**
(Order does not matter; record the number of correct words repeated.)
Correct words: [____]

**Internet use:**

**A1. Do you use the internet? (Including accessing the internet via mobile phones and other electronic devices)**

Use the internet every day .............................................................. 1

Use the internet at least once a week ............................................. 2

Use the internet at least once a month ........................................... 3

Use the internet a few times a year ................................................ 4

Never use the internet .................................................................... 5

**A2. What device(s) do you primarily use to access the internet? (Multiple choices allowed)**

Mobile phone ................................................................................... 1

Computer ......................................................................................... 2

Tablet (Pad/iPad, etc.) ..................................................................... 3

Other internet-enabled devices (Please specify: ________) ........... 4

**A3. How proficient are you in using these devices?**

|  | Not proficient at all | Not very proficient | Average | Quite proficient | Very proficient |
| --- | --- | --- | --- | --- | --- |
| a. Mobile phone | 1 | 2 | 3 | 4 | 5 |
| b. Computer | 1 | 2 | 3 | 4 | 5 |
| c. Tablet (Pad/iPad, etc.) | 1 | 2 | 3 | 4 | 5 |
| d. Other internet-enabled devices | 1 | 2 | 3 | 4 | 5 |

**A4. What do you mainly use the internet for? (Multiple choices allowed)**

Voice or video chats ...................................................................... 1

Text messaging .............................................................................. 2

Online shopping ............................................................................ 3

Reading news ................................................................................ 4

Browsing non-news articles/information ...................................... 5

Listening to music/radio, watching videos ................................. 6

Playing games ............................................................................... 7

Transportation and travel ............................................................. 8

Health management ....................................................................... 9

Financial investments (e.g., stock trading, buying funds) .......... 10

Learning and training .................................................................... 11

Other (Please specify: ____________) ....................................... 12

**Social networks:**

|  | none | 1 | 2 | 3-4 | 5-8 | 9 and above |
| --- | --- | --- | --- | --- | --- | --- |
| 1. How many family members/relatives do you meet or contact at least once a month? |  |  |  |  |  |  |
| 1. How many family members/relatives can you confide in about personal matters? (Cannot be answered on behalf of the respondent) |  |  |  |  |  |  |
| 1. How many family members/relatives can provide help when you need it? |  |  |  |  |  |  |
| 1. How many friends do you meet or contact at least once a month? |  |  |  |  |  |  |
| 1. How many friends can you confide in about personal matters? (Cannot be answered on behalf of the respondent) |  |  |  |  |  |  |
| 1. How many friends can provide help when you need it? |  |  |  |  |  |  |

**Depressive symptoms:**

1. **In the past week, did you feel in a good mood?**

Never.............................................................................. 1

Sometimes...................................................................... 2

Often.............................................................................. 3

Cannot answer................................................................ 9

1. **In the past week, did you feel lonely?**

Never.............................................................................. 1

Sometimes...................................................................... 2

Often.............................................................................. 3

Cannot answer................................................................ 9

1. **In the past week, did you feel very sad?**

Never.............................................................................. 1

Sometimes...................................................................... 2

Often.............................................................................. 3

Cannot answer................................................................ 9

1. **In the past week, did you feel like your life was going well?**

Never.............................................................................. 1

Sometimes...................................................................... 2

Often.............................................................................. 3

Cannot answer................................................................ 9

1. **In the past week, did you feel like you didn't want to eat?**

Never.............................................................................. 1

Sometimes...................................................................... 2

Often.............................................................................. 3

Cannot answer................................................................ 9

1. **In the past week, did you have trouble sleeping?**

Never.............................................................................. 1

Sometimes...................................................................... 2

Often.............................................................................. 3

Cannot answer................................................................ 9

1. **In the past week, did you feel like you were useless?**

Never.............................................................................. 1

Sometimes...................................................................... 2

Often.............................................................................. 3

Cannot answer................................................................ 9

1. **In the past week, did you feel like you had nothing to do?**

Never.............................................................................. 1

Sometimes...................................................................... 2

Often.............................................................................. 3

Cannot answer................................................................ 9

1. **In the past week, did you feel like there were many enjoyable things in your life?**

Never.............................................................................. 1

Sometimes...................................................................... 2

Often.............................................................................. 3

Cannot answer................................................................ 9
